# Investigation of the Aggregation of Aβ Peptide (1-40) in the Presence of κ-Carrageenan-Stabilised Liposomes Loaded with Homotaurine

**DOI:** 10.3390/molecules29153460

**Published:** 2024-07-24

**Authors:** Kamelia Kamburova, Ivaylo L. Dimitrov, Feyzim Hodzhaoglu, Viktoria Milkova

**Affiliations:** Institute of Physical Chemistry ‘Acad. R. Kaischew’, Bulgarian Academy of Sciences, 1113 Sofia, Bulgaria; kamelia@ipc.bas.bg (K.K.); idimitrov@ipc.bas.bg (I.L.D.); feyzim@ipc.bas.bg (F.H.)

**Keywords:** amyloid β peptide, homotaurine, κ-carrageenan, liposomes, aggregation, marine drugs

## Abstract

The kinetics of amyloid aggregation was studied indirectly by monitoring the changes in the polydispersity of mixed dispersion of amyloid β peptide (1-40) and composite liposomes. The liposomes were prepared from the 1,2-dioleoyl-sn-glicero-3-phoshocholine (DOPC) phospholipid and stabilised by the electrostatic adsorption of κ-carrageenan. The produced homotaurine-loaded and unloaded liposomes had a highly negative electrokinetic potential and remarkable stability in phosphate buffer (pH 4 and 7.4). For the first time, the appearance and evolution of the aggregation of Aβ were presented through the variation in the standard percentile readings (D10, D50, and D90) obtained from the particle size distribution analysis. The kinetic experiments indicated the appearance of the first aggregates almost 30 min after mixing the liposomes and peptide solution. It was observed that by adding unloaded liposomes, the size of 90% of the particles in the dispersion (D90) increased. In contrast, the addition of homotaurine-loaded liposomes had almost minimal impact on the size of the fractions of larger particles during the kinetic experiments. Despite the specific bioactivity of homotaurine in the presence of natural cell membranes, this study reported an additional inhibitory effect of the compound on the amyloid peptide aggregation due to the charge effects and ‘molecular crowding’.

## 1. Introduction

The neurodegenerative brain disorder (NBD) is a common term for the processes of progressive loss of the structure or function or the death of neurons resulting in the progressive loss of the ability to learn, mental, behavioural, or functional decline, or the gradual loss of emotions and movements of the body. Alzheimer’s disease (AD) is a more representative NBD. The World Health Organization (WHO) estimates that approximately 55 million people (2019) aged 65 years or older have AD diagnosis, and additionally, about 4.6 million new cases are registered every year. The number of cases is expected to increase to 66 million in 2030 and to 139 million in 2050 [1].

There are few theories (hypotheses) for the presumable molecular and pathophysiological mechanisms for the explanation of the manifestation and the progression of the disease [2]. Among them, the most well-supported is ‘the amyloid cascade hypothesis’ (ACH). The Aβ peptide (Aβ) is a short segment of the transmembrane amyloid precursor protein (APP) released after abnormal cleavage by the enzymes β- and γ-secretase. The Aβ molecules can aggregate to form low-molecular weight flexible and soluble oligomers, which may coalesce to form high-molecular weight oligomers, with β-sheet conformation, protofibrils, and insoluble fibrils deposited in diffuse senile plaque, which disturbs the normal function of neurons and is a characteristic feature of AD.

The presence of the soluble aggregates of amphiphilic proteins that can undergo evolution in ordered fibrils is considered a main hypothesis and common feature in the pathology of diseases related to NBD. Concerning AD, many postmortem observations indicate that the disease is characterised by the extracellular deposition of fibrous aggregates or intracellular inclusions containing Aβ peptide in the brain parenchyma and cerebral blood vessels of the patients.

Amyloid aggregation and fibrillation have been theoretically described using a nucleation-dependent polymerisation process [3,4,5]. The model was presented with two phases—nucleation and extension and secondary nucleation steps. The formation of the nucleus results from a series of association steps of Aβ monomers. These interactions are thermodynamically unfavourable, and this stage was determined as the rate-limiting step in fibril formation. The transition of monomer to fibril is a multi-step process. The energy barrier of the primary nucleation step is higher compared to the energy barrier for the incorporation of the new monomers in the existing protofibrils or fibrils [6]. Fibril growth has been described using a ‘dock-lock’ model. According to that model, the monomers interact rapidly with the existing fibril ends (‘dock’), followed by a conformational change to find the more thermodynamically favourable and stable state of the peptide (β-sheet conformation) (‘lock’) [7]. The variation in the pH, temperature, ionic strength, or presence of cell membrane can affect the aggregation process.

Currently, there is no effective therapy for AD. The existing therapeutic strategies are based on the presumable pathophysiological mechanisms of the disease, and they can only ameliorate the suffering of the patients, but they do not cure. At present, there are several active molecules approved by the US Food and Drug Administration (FDA) for the treatment of AD—tacrine, donepezil, rivastigmine, galantamine, and memantine.

Concerning the neuroprotection activity of the natural compounds, the substances extracted from marine organisms possess a promising biotechnology potential. More than 10,000 natural components have been isolated from bacteria, fungi, algae, and other marine organisms. There are a significant number of extracted bioactive molecules (chitosan, fucoidan, homotaurine, gracillin, caniferolides, bryostatin-1, dictyostatin, anabasine, rifampicin, etc.) with potential application in the therapy of NBDs [8,9].

Homotaurine (HT, 3-amino-1-propanesulfonic acid or Tramiprosate) is a natural amino acid extracted from red, green, or brown algae. It has been also found in marine roseobacters. The therapeutic properties of HT have been tested in a few phase II and phase III clinical studies, and it has been confirmed that HT decreases the concentration of soluble Aβ in the cerebrospinal fluid (SCF) and affects the deposition of amyloid fibrils in a plaque in the brain [10]. According to a presumable mechanism of therapeutic action, the molecules of HT are BBB-permeable and can interact with the peptide to prevent (limit) the formation of neurotoxic Aβ oligomers and prevent the misfolding processes that lead to amyloid aggregation and cause neurotoxicity and progression in AD [11,12,13,14].

Homotaurine is classified as a compound that mimics glycosaminoglycan (GAG), which binds to beta-amyloid peptide molecules and thus facilitates their fibrillation. In the competitive scenario, the compound has an affinity for the GAG-binding region of Aβ molecules and thereby displaces GAGs, reduces fibrillation, and reverses the conformational changes in Aβ molecules that lead to amyloid plaque deposition [10,12].

κ-carrageenan (CAR) is a polysaccharide, a product of extraction from certain species of red seaweeds. It is a linear, water-soluble polymer that typically forms highly viscous aqueous solutions. Moreover, recently, it has been reported that the oligosaccharides of κ-carrageenan postpone the progression of AD by preventing damage to the neurons [15].

The present study is focused on the experimental investigation of the effect of the presence of homotaurine-loaded liposomes on the kinetics of Aβ aggregation.

The liposomes were chosen for this study because they are drug delivery platforms with many advantages. They are non-toxic and have a high drug-loading capacity. Concerning their application in neurological studies, they are extremely promising delivery platforms because nano-sized liposomes could cross the blood–brain barrier (BBB) [16].

In this study, the appearance and evolution of the amyloid aggregation in the presence and absence of liposomes were presented through the variation in the standard percentile readings (D10, D50, and D90) obtained from the particle size distribution analysis from kinetic experiments. The percentiles are statistical terms that correspond to the fraction of particles with a size equal to or less than a certain value. For intensity-weighted particle size distributions, the parameters are reported based on the maximum particle size for a given percentage intensity of the sample. The standard percentile readings from the analysis are denoted as D10, D50, and D90, where D stands for diameter, and 10, 50, and 90 represent the percentage of the sample below a certain particle size (e.g., 10%, 50%, and 90%). D50 represents the median of the intensity distribution and is defined as the particle size at which half of the population lies below this value. By monitoring these three parameters in the kinetics experiments, it is possible to observe any significant changes in the main particle size and changes at the extremes of the distribution, which could be due to the presence of fines or oversized particles/agglomerates.

The novelty of the present work is addressed to the investigation of amyloid aggregation in the mixture dispersion of peptide molecules and composite liposomes loaded by anti-amyloid agents. Moreover, all components in the system are completely natural compounds extracted from marine organisms (excluding lipid and amyloid peptides). The appearance of amyloid aggregation in conditions close to the physiological ones was indirectly investigated by monitoring the variation in the polydispersity of the species in dispersion.

## 2. Results

### 2.1. Characterisation of the HT-Loaded Liposomes

Table 1 presents the experimental data for the hydrodynamic size and ζ-potential of the produced unloaded and HT-loaded liposomes.

**Figure A1:**
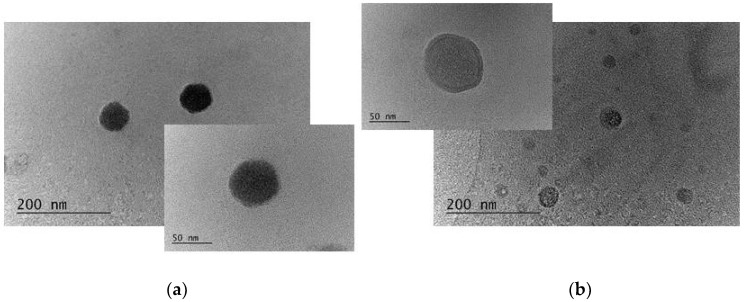
Visualisation of the produced liposomes: (**a**) unloaded and (**b**) HT-loaded.

According to the presented results, the values of size and charge of the HT-loaded liposomes are higher than those of the unloaded ones. One possible explanation is a variation in the liposome structure and the incorporation of some HT molecules in the lipid bilayer during the encapsulation process. In support of this assumption is the registered very high encapsulation efficiency of the compound in the liposomes (ca. 99%). The HT molecules have a slightly negative charge, and their incorporation in the bilayer leads to an increase in the net negative charge of the liposomes. When negatively charged carrageenan molecules are adsorbed onto liposomes, they stabilise them, forming a thick polymer layer (about 18 nm) and increasing their net negative charge. A visualisation of the produced liposomes (unloaded and HT-loaded) is presented in Figure A1 (Appendix A).

### 2.2. Estimation of the Amount of HT Encapsulated into the Liposomes

A spectrophotometric assay is applied to evaluate the quantity of loaded HT in the liposomes. In the applied methodology, methylene blue is used as an oxidant. Methylene blue exhibits redox properties, appearing blue in its oxidised form and colourless in its reduced form. Under acidic conditions, the methyl groups of the compound preferentially dissociate due to the protonation of the dimethylamino group [17,18]. When methylene blue reacts with HT in an acidic medium, its absorbance value gradually decreases. By using a suitable calibration curve, the entrapped amount of HT in the liposomes is extremely high (ca. 0.099 mg/mL), and the estimated encapsulation efficiency is ca. 99%.

### 2.3. Kinetics of Aggregation of Aβ at Different pH and Temperatures

**Figure 1:**
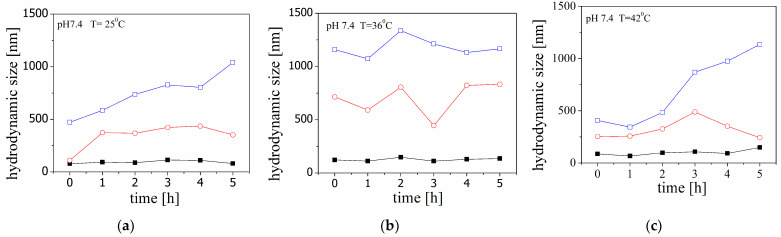
A variation in the hydrodynamic size (diameter) of Aβ peptide at pH 7.4 in the kinetic experiments at 25 °C (**a**), 36 °C (**b**), and 42 °C (**c**). The size is presented through the percentiles in the size distribution D10 (■), D50 (○), and D90 (□).

**Figure 2:**
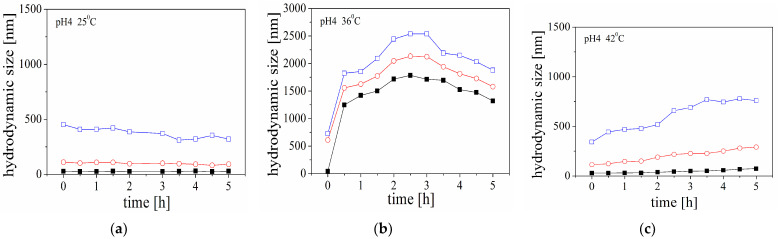
A variation in the hydrodynamic size of Aβ peptide at pH 4 in the kinetic experiments at 25 °C (**a**), 36 °C (**b**), and 42 °C (**c**). The size is presented through the percentiles in the size distribution D10 (■), D50 (○), and D90 (□).

**Figure A2:**
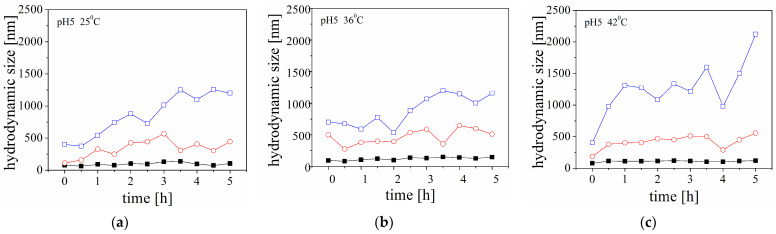
A variation in the hydrodynamic size (diameter) of Aβ peptide at pH 4 in the kinetic experiments: (**a**) at 25 °C, (**b**) 36 °C, and (**c**) 42 °C. The size is presented through the percentiles in the size distribution: D10 (■), D50 (○), and D90 (□).

The changes in the size of the peptide molecules in solution at pH 7.4 and pH 4 with an increase in temperature are illustrated by standard percentiles in Figure 1 and Figure 2 (the experimental results for the system at pH 5 are presented in Figure A2, Appendix A). The figures present results from three samples measured at three different temperatures.

**Figure 3:**
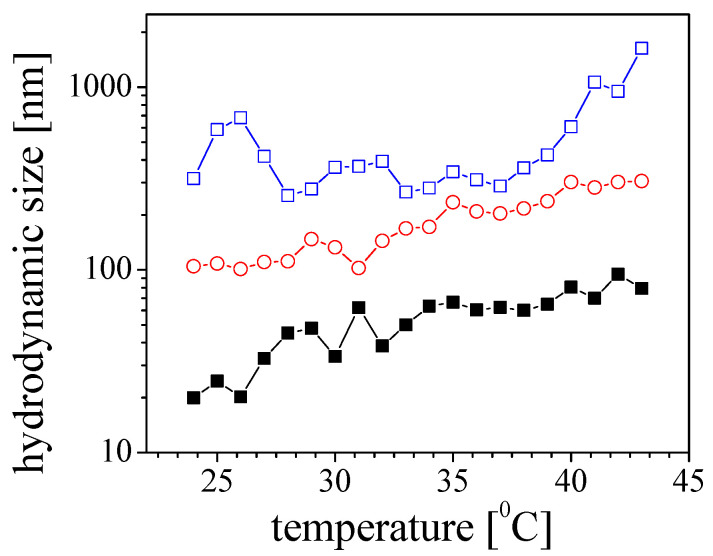
A variation in the hydrodynamic diameter of Aβ peptide at pH 4 in a stepwise regime of increasing the temperature. The size is presented through the percentiles in the size distribution D10 (■), D50 (○), and D90 (□).

The findings of the kinetic experiment indicated that a notable aggregation process was observed when the temperature was 36 °C and the pH was 4. Interestingly, the emergence of structures with a size of approximately 2000 nm was observed within the first 30 min (Figure 2b). On the other hand, when the sample is subjected to a gradual temperature increase in the cell (Figure 3), the size values and their dependencies take on a different shape. The structures in the samples were observed to gradually increase in size. This suggests distinct aggregation mechanisms induced by temperature, which may explain the results obtained.

### 2.4. Kinetics of Aggregation of Aβ in the Presence of Homotaurine

**Figure 4:**
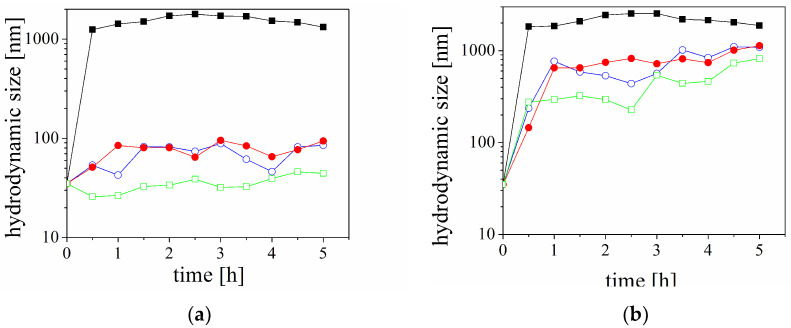
A variation in the hydrodynamic diameter of pure Aβ peptide (■) and mixtures of peptide and the following different concentrations of homotaurine: 0.05 mg/mL (○), 0.1 mg/mL (●), and 0.5 mg/mL (□). The size is presented through the percentiles in the size distribution D10 (**a**) and D90 (**b**). The pH of the dispersions is 4.

The aggregation behaviour of peptides in the presence of homotaurine is presented in Figure 4. The experimental results indicate significantly lower values of the size of the fraction of particles D10 and D90 in the presence of homotaurine compared to the distribution in a pure solution of Aβ.

### 2.5. Kinetics of Aggregation of Aβ in the Presence of Liposomes

**Figure 5:**
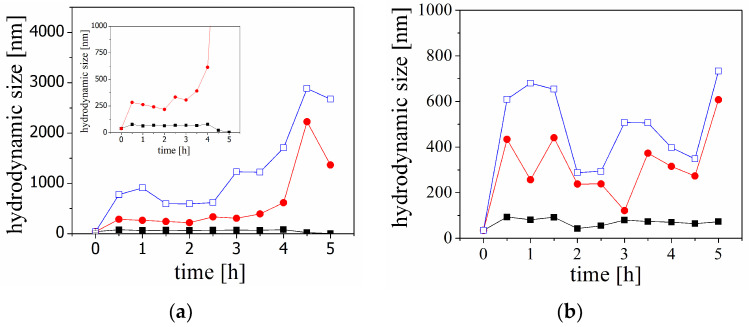
A variation in the hydrodynamic size of the Aβ in the presence of unloaded (**a**) and HT-loaded liposomes (**b**). The size is presented through the percentiles in the size distribution D10 (■), D50 (○), and D90 (□). The inset presents the expanded dependence of the percentiles D10 and D50.

Figure 5 illustrates the percentiles (D10, D50, and D90) in the size distribution of particles in a solution of Aβ during kinetic experiments in the presence of unloaded or HT-loaded liposomes. The results suggest that larger particles or aggregates dominate, and their size increases with reaction time. The decrease in the size of fraction D90 at the last measurement (almost 5 h after mixing the peptide solution and liposomes) is likely due to the sedimentation of the largest particles in the experimental cell. Additionally, the values of the size of the fraction D50 decrease, indicating that disruption of the lipid bilayer is negligible.

The addition of the HT-loaded liposomes does not indicate significant aggregation during the kinetic experiments. The first change in the size distribution was observed about 30 min after mixing the peptide solution and liposomes, and the sizes corresponding to percentiles D10, D50, and D90 were almost constant until the end of the experiment.

**Figure 6:**
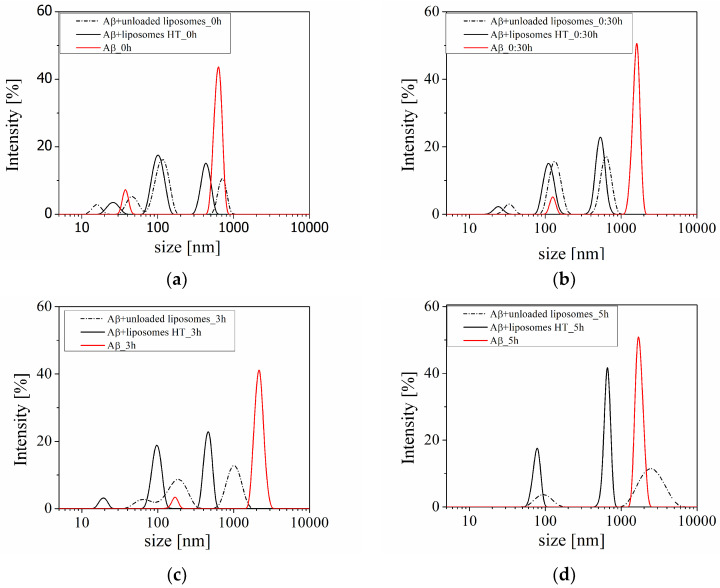
The size distribution during the kinetic measurements at pH 4 in pure Aβ peptide solution (red line), solution of the peptide in the presence of unloaded (dash line), and HT-loaded liposomes (solid black line) immediately after mixing the solutions (**a**), 30 min (**b**), 3 h (**c**), and 5 h (**d**).

The raw data from the DLS measurements are more representative. In Figure 6, the shift of the peaks during the kinetics experiment can be observed.

## 3. Discussion

The liposomes as delivery platforms suggest many possibilities and advantages for application in successful therapies for NBDs. Some studies have reported that small liposomes (up to 100 nm) can pass through the BBB and deliver drugs or bioactive molecules, with potential activity against amyloidosis in the brain. However, the presence of liposomes, as a model of biological membranes, can also induce the aggregation of amyloid peptides.

According to ACH, the critical micromolar concentration of free peptide monomers is required to form the first oligomers. However, postmortem observations have shown that the amyloid concentration found in the brain is exceptionally lower, and the probability for the observation of a spontaneous oligomerisation of Aβ at the estimated pico- or nanomolar peptide concentration is very low. The observed limitation in the ACH has been explained with the participation of additional effects on the aggregation kinetics as Aβ–membrane interactions.

It has been previously reported that the interaction of peptides with the cell membrane (or the surface of a liposome in the model studies) is governed not only by the physicochemical characteristics of the membrane (size or the curvature, composition, elasticity, charge, degree of hydration, conformation, and dynamics of lipid headgroups) but also by the thickness of the hydrophobic part of the bilayer or surface modification of the membrane, the pH of the medium, or the presence of metal ions in the solution [19,20].

In the present study, in order to elucidate the influence of the liposomes and HT on the aggregation, kinetic experiments were performed with the peptide solution mixed with a solution of homotaurine at acid conditions (pH 4). The experimental results indicate that the Aβ peptide aggregation is increasingly suppressed with the increasing HT concentration in the system (Figure 5). The results indicate significantly lower values of the size of 90% of the particles in the solution (D90) in the presence of the homotaurine compared to the distribution in a pure solution of Aβ at the same experimental conditions. The peptide molecules are positively charged at the experimental conditions (ζ-potential is ca. + 10 mV). The addition of HT (weak sulfonic acid) does not affect the ionic strength or pH of the solution. Therefore, it was expected that attractive electrostatic interactions or specific attractive interactions between the peptide and homotaurine are governed by the very complicated structure of Aβ (hydrogen bonding or hydrophobic interactions). Additionally, as the concentration of HT increases, the total concentrations of species in solutions increase, leading to ‘molecular crowding’. This phenomenon can also inhibit the aggregation process.

Postmortem observations have shown that the pH of the brain parenchyma is lower for patients with an Alzheimer’s diagnosis (<pH 4). Furthermore, experimental studies have confirmed that the variation in pH is a key factor for amyloidosis [21,22]. Therefore, a detailed screening of the conditions in model experiments can ensure optimal conditions for investigations of the mechanisms of the Aβ peptide aggregation.

The present study reports preliminary investigations on the kinetics of aggregation of Aβ peptide in the presence of unloaded and HT-loaded liposomes. The electrostatic interactions between the positively charged peptide monomers and zwitterionic liposomes are additionally affected by the adsorption of carrageenan. It was presumed that the behaviour of Aβ peptide in the presence of highly negatively charged polymer-coated liposomes would be fundamentally different compared to the properties of pure peptide solution or a dispersion of carrageenan-free liposomes. Experimental data for the time dependence of the sizes of 90% of the particles in the dispersion (D90) indicate that a significant part of the peptide molecules is involved in aggregate formation (Figure 1 and Figure 2).

According to the results from the kinetic experiments, the size of the fractions of species D10, D50, and D90 in the mixtures of peptide and liposomes suggest that the larger particles or aggregates dominate, and their size increases with reaction time (Figure 5).

The time dependence of the particle size in the solution of Aβ peptide in the presence of unloaded liposomes can be divided into two stages (Figure 5a). An increase in the size is registered in the first stage, and the beginning of the aggregation is observed 30 min after mixing the peptide solution and the liposomes. After that, the size of 90% of the particles in the dispersion (D90) gradually increases up to 3 h. Meanwhile, the fractions of aggregates are significantly larger compared to the fraction of smaller particles and increase with time. For example, two hours after mixing, the size of 90% of the particles (D90) is ca. 600 nm, the size of 50% of the particles (D50) is ca. 200 nm, and the smallest particles are ca. 70 nm. In the second stage, sharp increases in the size of larger particles are observed. The fraction of the smaller particles is almost constant during the measurement (D10).

The time dependence of Aβ aggregation in the presence of HT-loaded liposomes shows different kinetic behaviour (Figure 5b). The aggregation in the dispersion is also registered 30 min after the mixing, and the size of 90% of the particles (D90) is ca. 700 nm. However, in the presence of HT-loaded liposomes, the size of the fractions D50 and D90 almost do not change during the kinetic experiment.

The analysis of the peaks in the size distribution displayed in Figure 6 indicates that the peaks have shifted throughout the experiment. Notably, the positioning of the peaks in the most recent measurements is particularly intriguing (Figure 6d). There are two peaks registered from the dispersion of peptides in the presence of unloaded liposomes. One peak is at the position of Aβ, similar to the pure peptide solution but with lower intensity, while the other peak corresponds to the smallest particles in the system. In the dispersion containing HT-loaded liposomes, there are also two peaks, but they correspond to particles with smaller sizes than the amyloid peptide aggregates. This experimental result clearly illustrates the effect of the HT-loaded liposomes on amyloid aggregation.

The observed kinetic behaviour is in line with previously reported mechanisms of amyloid aggregation in the presence of a membrane. According to the presented models, the aggregation process can be described as a sigmoidal function of time on the fraction of aggregates. The initial nucleation phase (lag phase) is a relatively slow process of the formation of first aggregates, whereas the sequential elongation phase is rapid [23]. However, aggregation is a very sensitive process, and variations in the conditions can promote or hinder the aggregation.

Previously, it has been reported that the presence of a membrane may induce conformational changes in the amyloid molecules, and the formation of a partially unfolding molecular structure has been suggested as a critical step in fibrillation [24]. The conformational changes have been interpreted as a result of the local accumulation of protons in the vicinity of the negatively charged lipid membrane. This local decrease in pH promotes an increase in the peptide charge density and repulsive electrostatic interactions with the membrane. The protein molecule becomes more open, and the hydrophobic parts are more favourable for aggregation. Therefore, the charge of the membrane is a key factor in the structural transition of the peptide molecules. It has been shown that in the case of Aβ peptide, the fibrillation in the presence of anionic and zwitterionic membranes is governed predominantly by charge effects instead of conformation changes. These membranes promote aggregation at low peptide concentrations [25].

At a low pH, Aβ peptide is negatively charged, and attractive electrostatic interactions with positively charged domains on the zwitterionic membranes will occur. As a result, the increase in a local peptide concentration on the membrane is observed. Wilson and Binder [26] have proposed that the high local peptide concentration overcomes the energy barrier for nucleation and promotes aggregation.

Following these hypotheses, we supposed that the surface modification and variation in the electrical properties of the lipid membrane could enhance, promote, or hinder peptide aggregation. At the experimental conditions of a pH of 4, the DOPC liposomes have an almost neutral net charge [27]. The sequential adsorption of negatively charged carrageenan on the positively charged domains leads to an increase in the net negative charge of the liposomes. At the same time, the peptide molecules are positively charged at a pH of 4 (the estimated pI of Aβ (1-40) peptide is 5.4 [28]). Thus, it is expected that the adsorption of CAR would promote the electrostatic interaction between the liposomes and the peptides and peptide aggregation, respectively. However, the comparison between the aggregation behaviour in the solutions of pure Aβ peptide and the aggregation in the dispersions of unloaded liposomes and Aβ peptide (Figure 2b and Figure 5a) indicates that the aggregation is suppressed in the presence of the liposomes. While the maximal size of 90% of the particles (D90) is approximately 2600 nm for the solution of Aβ and 3000 nm for the mixture, the processes of aggregation kinetic are different. The retardation time (ca. 3 h) was observed for the solution containing liposomes.

Under the same experimental conditions, the negative charge density of the HT-loaded particles is higher than that of the unloaded ones. We supposed that this results from the incorporation of a compound in the lipid layer. The sequential polymer adsorption leads to an additional increase in the negative charge of the liposomes, and contrary to the expectation, the peptide aggregation is hampered.

To explain the obtained results, we supposed that there were additional factors that influenced the process. Firstly, the ionic strength of the solution determined by the buffer is extremely high (ca. 10^−1^ M). Therefore, a significant screening of the electrostatic interactions between the peptide molecules and the liposomes can be expected. Secondly, CAR molecules are fully charged at the experimental conditions (pH 4). Along with the expected charge renormalisation in the CAR polyion vicinity (‘counterion condensation’), the additional charge effects related to the presence of high salt concentration in the solution must be considered in the screening of the electrostatic interactions [29,30]. Thirdly, the polymer layer is formed at low ionic strength (10^−4^ M). According to the theory of strong polyelectrolytes, the adsorption layer has to be thin due to the strong electrostatic repulsion between the charges along the polyion chain. However, it is well known that carrageenans are high-molecular weight polysaccharides, and the formation of loops and tails is expected in the adsorption layer (the thickness of the CAR layer is ca. 18 nm, Table 1). Hence, the participation of additional steric interactions could be responsible for hampering the aggregation process of Aβ peptides.

Many questions remain open in this study. As far as we know, this is the first complex study of the aggregation of Aβ peptide in the presence of polymer-modified liposomes and co-encapsulated bioactive molecules. The experimental results indicate that the adsorption of polymers on the liposomes influences the peptide aggregation, but the effect of the charge density is still unclear. For this purpose, a zwitterionic membrane would be a useful model surface. Homotaurine suppresses the aggregation of the Aβ 1-40 peptide on the liposomes, but the nature of the mechanism of interaction is also unknown. Additional experimental methods have to be applied for the characterisation of the charge effects in the presence of proteins or other polyelectrolytes. A detailed visualisation of the aggregates (from oligomers to protofibrils or fibrils) under the used experimental conditions is also needed and will be the subject of another study. The distinction of the fraction of aggregates from peptide molecules or peptide–liposome complexes in the mixtures is complicated using only electrokinetic methods.

## 4. Materials and Methods

### 4.1. Materials

The phospholipid 1,2-dioleoyl-sn-glicero-3-phoshocholine (DOPC, chloroform solution, 25 mg/mL) product of Avanti Polar Lipids Inc. (Alabaster, AL, USA) was used for the preparation of unilamellar liposomes.

Amyloid beta, Aβ (1-40), was purchased from Sigma Aldrich (St. Louis, MO, USA, product number SCP0037-0.5 mg). A stock peptide solution was prepared as follows: to the lyophilised commercial peptide sample, NH_4_OH was added to obtain a 12.5 mg/mL peptide concentration (the solution of 1% NH_4_OH was filtered before use through 0.22 µm filters (Minisart^®^, Sartorius, Gottingen, Germany) to eliminate eventual dust particles). The peptide solution was then gently diluted (without vortex) to a concentration of 0.8 mg/mL with 150 mM PBS buffer with a pH of 7.4. The buffer solution was also filtered before use. It was prepared using a standard recipe with NaCl (137 mM), KCl (2.7 mM), Na_2_HPO_4_ (10 mM), and KH_2_PO_4_ (1.8 mM). The dissolved protein solution was separated into 40 aliquots of 15 µL and stored at −20 °C until use. The final concentration of the peptide in the experiments was 0.4 mg/mL, corresponding to 10 µM. For the electrokinetic measurements, the samples were further diluted with buffer to a final peptide concentration of 0.016 mg/mL.

k-carrageenan (CAR) and homotaurine (HT) were also purchased from Sigma Aldrich. k-carrageenan (product number 22048) was used in this study without any purification. The stock solutions with a concentration of 2 mg/mL were prepared in double-distilled water. The prepared polymer solutions were filtered through a 5 µm filter (Minisart^®^, Sartorius) to remove the possible aggregates.

### 4.2. Methods

#### 4.2.1. Liposome Preparation

The liposomes were prepared by using the thin film hydration method. The procedure was described in detail in a previous study [31]. Briefly, an appropriate volume (200 µL) from the solution of lipid in chloroform (25 mg/mL) was dried under a stream of nitrogen by rotating the glass flask to form a thin lipid film on its wall.

To produce unloaded or HT-loaded liposomes, the lipid was rehydrated in 2 mL of double-distilled water or a solution of HT (0.1 mg/mL) to a final lipid concentration of 2.5 mg/mL. After 4 freeze–thaw cycles of the tube with dispersion in a bath of liquid nitrogen and tap water, the stock solution of liposomes was sonicated in an ultrasonic ice bath for 15 min.

In order to prevent a possible aggregation during the subsequent steps in the experimental procedure, the work dispersion was prepared by mixing 80 µL from the stock dispersion of liposomes and 10 mL of double-distilled water to a final lipid concentration of 0.02 mg/mL. The dispersion was filtered before measurements by extrusion through a 0.20 µm filter (Minisart^®^, Sartorius).

To improve the stability of the loaded liposomes, a carrageenan monolayer was adsorbed on their surfaces. The polymer layer was formed by adding a diluted dispersion of liposomes (9.5 mL) to the solution of negatively charged CAR (0.5 mL, 2 mg/mL) and stirring for 20 min.

#### 4.2.2. Kinetic Experiments

The aggregation kinetics of Aβ was investigated in the following four different experiments:

- Investigation of the effect of pH.

The samples for the measurement were prepared by adding 15 µL of phosphate buffer with a pH of 4, 5, or 7.4 (adjusted with HCl) to the Eppendorf tube containing 15 µL of peptide solution. The final concentration of peptide was 0.4 mg/mL (10 µM). The measurements were performed by adding 25 µL of the solution to a micro cell for DLS. The variation of the size distribution of the peptide aggregates was evaluated at 24 °C, 36 °C, and 42 °C.

- Effect of the temperature at a pH of 4.

The samples were prepared similarly, as follows: 15 µL of phosphate buffer with a pH of 4 (adjusted with HCl) was added to the Eppendorf tube containing 15 µL of peptide solution. The final concentration of peptide was 0.4 mg/mL. The measurements were performed over a large temperature interval (24–43 °C) in a stepwise manner. The interval between the different values of the temperature was 10 min. Three minutes of this time interval were used for the sample equilibration, and seven minutes were used for the measurement cycle.

- Aggregation of peptide in the presence of homotaurine at a pH of 4 and 36 °C.

The samples for these experiments were prepared similarly to those in the other experiments. Phosphate buffer (pH 4 adjusted with HCl) was mixed with homotaurine (powder). The final concentrations of HT in the solutions were 0.05, 0.1, and 0.5 mg/mL.

- Aggregation of peptide in the presence of unloaded or HT-loaded liposomes.

The dispersions of liposomes stabilised by the adsorption of carrageenan (lipid concentration of 6 × 10^−4^ mg/mL in distilled water) were centrifuged (15,000 rpm at 19 °C for 90 min) using a laboratory centrifuge (PW-352R, Warsaw, Poland). After centrifugation, the liposomes were settled, and the supernatant was completely removed. The liposomes were redispersed in a peptide solution using sonication (5 min) in an ultrasonic ice bath. Then, 25 µL from the dispersion was added to a microcell for DLS, and 15 µL of HCl (0.1 M) was added to the dispersion. The measurements were performed at 36 °C and a pH of 4 (the peptide samples were prepared by adding 15 µL of phosphate buffer (pH 7.4) to the Eppendorf tube containing 15 µL of peptide solution).

#### 4.2.3. Determination of the Electrokinetic Charge and Particle Size Distribution

During the experimental procedure, the size and charge of the produced liposomes were assessed using dynamic light scattering with non-invasive backscattering (DLS-NIBS) at a measuring angle of 173°. The Zetasizer Pro (Malvern, UK), equipped with a He-Ne laser with a maximum power of 10 mW and operating at a wavelength of 633 nm, was used to carry out the measurements. The liposomes were measured five times, and the average value was recorded as the final size and surface charge.

The kinetic measurements were performed using Zatasizer Pro with a low-volume quartz batch cuvette (Ultra-Micro Cell for Nano Series, with a minimal volume of 12 µL and a maximum volume of 45 µL, the width of the measurable window is 3 mm.). In the experiments, the percentiles in the particle size distributions of D10, D50, and D90 were evaluated.

#### 4.2.4. Determination of the Encapsulated Amount of Homotaurine in the Liposomes

The concentration of HT loaded in the liposomes was estimated using a spectrophotometric assay according to the procedure proposed by Zhao et al. [17]. Briefly, the samples were prepared as follows: 1.5 mL (1 M) of sulfuric acid solution was mixed in two test tubes with 0.35 mL (0.25 mg/mL) of methylene blue solution. An HT solution with a certain concentration was added to one of the tubes, and the tubes were diluted with distilled water to a final total volume of 10 mL. The reaction was carried out at room temperature for 20 min. The absorbances of the blank solution AU0 and AU of the solutions were measured at 664 nm, and the value of ΔAU = AU0 − AU was calculated. To obtain the calibration curve from HT, the differences ΔAU were calculated from the absorbance of 0 mg/mL HT (blank solution) and HT solutions with concentrations ranging from 10^−1^ to 10^−6^ mg/mL. The same procedure was applied to estimate the concentration of free HT in the dispersion of the liposomes. For this purpose, the stock dispersion was centrifuged at 15,000 rpm and 19 °C for 90 min, and the supernatant was extracted. The calculated difference ΔAU between the maximum absorption of the blank solution and supernatant corresponds to a free concentration of HT in the dispersion—ca. 5 × 10^−5^ mg/mL (evaluated from the calibration curve).

## 5. Conclusions

This study presents an investigation of the kinetics of amyloid peptide (1-40) aggregation by monitoring the changes in the polydispersity of mixed dispersions of the peptide and composite liposomes. The influence of the experimental conditions (pH, temperature) and the addition of different concentrations of HT and loaded and HT-loaded liposomes on the peptide aggregation was studied.

The aggregation of peptides was observed when the unloaded liposomes were added to the peptide solution, but the process was delayed. The estimated retardation time is approximately 3 h. Moreover, it was registered that in the presence of homotaurine-loaded liposomes, the aggregation process was significantly suppressed. To explain this phenomenon, it was presumed that there are additional factors that influence the process, as follows: screening of the electrostatic interactions between the peptide molecules and the liposomes due to the high ionic strength of the dispersions; charge renormalisation in the vicinity of the adsorbed fully charged carrageenan and the presence of counterions with lower mobility; and the participation of additional steric interactions due to the presence of a thick polymer layer (ca. 18 nm) on the liposomal surface.

## Figures and Tables

**Table 1:** Hydrodynamic diameter (D), polydispersity index (PDI), and electrokinetic potential of the liposomes. The concentration of CAR in stabilised dispersion was 0.1 mg/mL.

Sample	D * [nm] (PDI)	ζ-Potential [mV]
unloaded liposomes	37.1 ± 1.2 (0.12)	−55.1 ± 3.0
unloaded liposomes stabilised with CAR	52.2 ± 1.6 (0.11)	−62.7 ± 1.3
HT-loaded liposomes	55.6 ± 1.2 (0.22)	−59.8 ± 2.1
HT-loaded liposomes stabilised with CAR	92.1 ± 9.2 (0.19)	−65.0 ± 0.7

* Evaluated mean size by intensity.

**Figure A3:**
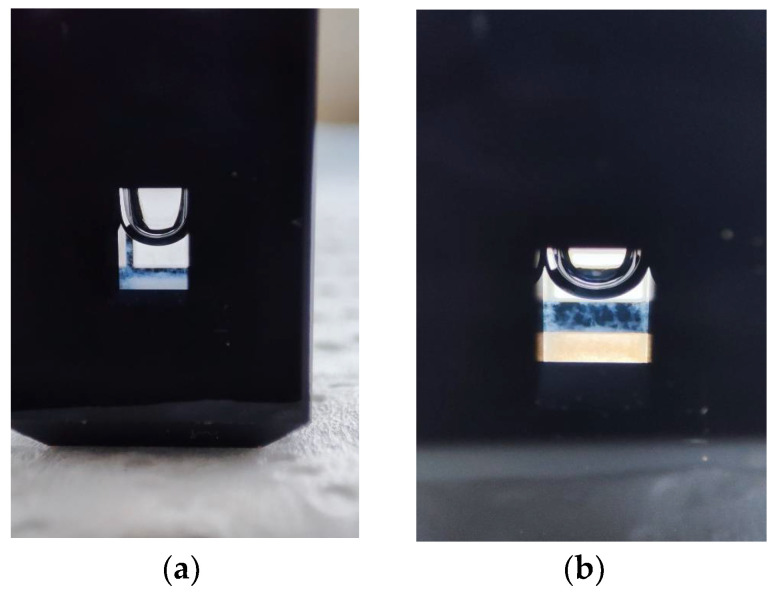
Image of the measuring cell for DLS with the sample after finishing the kinetic experiment: (**a**) observation of the aggregation of Aβ peptide in the cell at pH 4 and 36 °C. For DLS measurement, we used Ultra-Micro Cell for Nano Series. The width of the measurable window is 3 mm. (**b**) The same photo represented at a larger scale.

## Data Availability

Data are contained within the article.
